# Convexity subarachnoid hemorrhage presented with loss of consciousness

**DOI:** 10.1002/ccr3.1921

**Published:** 2018-11-22

**Authors:** Shinji Yamazoe, Yumi Okuyama, Akira Baba, Hiroyuki Yakabe, Yuko Kobashi, Takuji Mogami

**Affiliations:** ^1^ Department of Radiology Tokyo Dental College Ichikawa General Hospital Ichikawa Japan; ^2^ Department of Internal Medicine Tokyo Dental College Ichikawa General Hospital Ichikawa Japan

**Keywords:** computed tomography, convexity subarachnoid hemorrhage, transient loss of consciousness

## Abstract

When patients present with transient loss of consciousness without headache and head computed tomography is performed, clinicians should pay attention to cortical high densities as convexity subarachnoid hemorrhage can be a differential diagnosis.

A 91‐year‐old woman with a history of hypertension and diabetes mellitus presented to our emergency department with transient loss of consciousness. The patient was taking a bath alone and there was no witness. There was no sign of seizure when her family found her. She reported no headache or episode of trauma. Her blood pressure was 139/66 mm Hg, heart rate 90 bpm, body temperature 36.5°, and SpO2 97% (room air). The level was clear, and the symptom had disappeared before presentation. Physical examination revealed no neurologic finding. Her vital monitoring during examination kept sinus rhythm. The laboratory findings were significant for leukocytosis at 11 800/μL and hyperglycemia at 170 mg/dL. Head computed tomography (CT) revealed high densities and obscurations in left high‐convexity frontal sulci (Figure 1). Differential diagnoses included seizure, cerebral infarction, or bleeding. The location of bleeding as well as the fact that she experienced no headache led the diagnosis of convexity subarachnoid hemorrhage (cSAH). The patient was followed up by a neurologist and there were no remarkable findings. Two months after, the follow‐up head CT showed no high densities of left cortical frontal sulci or new lesions (Figure 2).

**Figure 1:**
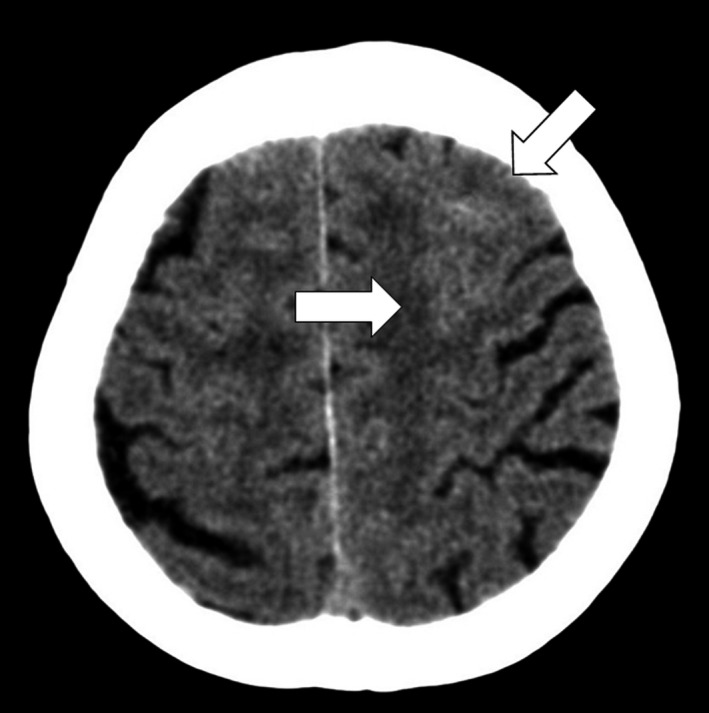
Head CT revealed high densities and obscurations in left high‐convexity frontal sulci (arrows)

**Figure 2:**
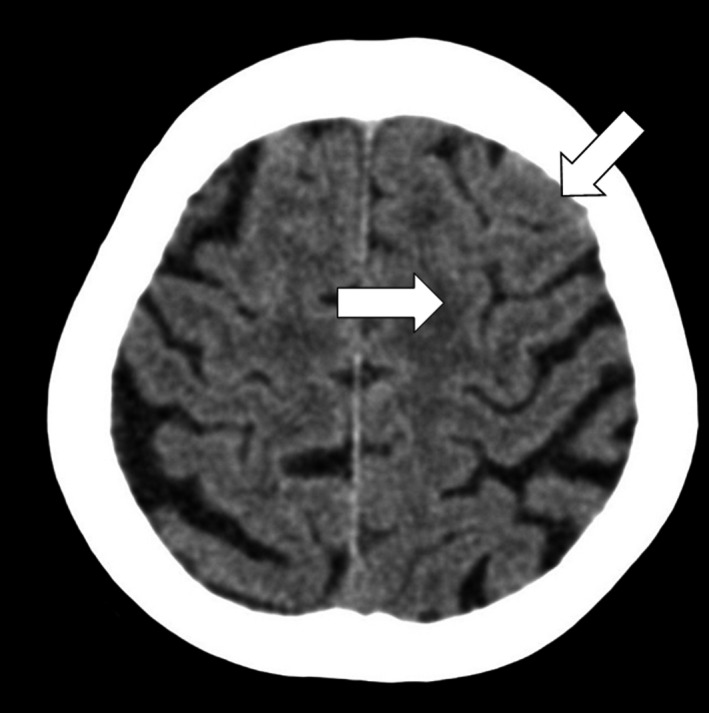
Head CT showed no high densities of left cortical frontal sulci or new lesions (arrows)

cSAH is rarely reported.^1^, ^2^ In cSAH, the most common symptom is focal neurologic deficits with transient loss of consciousness, but acute headache occurs in only about 20%‐40%.^1^, ^2^ When patients present with transient loss of consciousness and head CT is performed, clinicians should pay attention to cortical high densities as cSAH can be a differential diagnosis.

## CONFLICT OF INTEREST

None declared.

## AUTHOR CONTRIBUTION

SY: drafted the article. All authors participated in critical review and the revision of the article. All authors gave the final approval of the article. All authors have accountability for all aspects of the work.
